# Transillumination and Point-of-Care Ultrasonography to Delineate Tracheal Deviation for Challenging Tracheostomy: A Case Report

**DOI:** 10.7759/cureus.37164

**Published:** 2023-04-05

**Authors:** Prateek Arora, Ripudaman Arora, Habib Md R Karim, Praveen K Neema, Chinmaya K Panda

**Affiliations:** 1 Anaesthesiology, Critical Care, and Pain Medicine, All India Institute of Medical Sciences, Raipur, IND; 2 ENT and Head & Neck Surgery, All India Institute of Medical Sciences, Raipur, IND; 3 Cardiac Anaesthesiology, Amrita Institute of Medical Sciences and Research Centre, Kochi, IND

**Keywords:** fiberoptic bronchoscopy, tracheal deviation, tracheostomy procedure, percutaneous dilational tracheostomy, light detection, ultrasound-guided

## Abstract

Tracheostomy is indicated for varied reasons and is a relatively safe procedure that can be done with both open and percutaneous methods. However, the procedure is often challenging in cases of distorted neck anatomy. Neck swellings often push the trachea laterally or shield it. Even some gross intrathoracic pathology may shift the trachea from the typical trajectory making it challenging to delineate the course of the trachea. A bedside point-of-care technique having a visual aid that can guide the performer thus appears beneficiary. Fiber-optic assistance for correct puncture and confirmation is known, and light-based techniques have been used for tracheostomies. As fiberscopes are not infrequent in tertiary and even secondary care hospitals, transillumination from a flexible bronchoscope can identify the altered course of the trachea, much like a navigation system, and systematically aid the performer in steering away from the obstacles. We present two cases in two scenarios with tracheal deviation who underwent either open or percutaneous tracheostomy with point-of-care ultrasound and transillumination to delineate the course of the trachea and facilitate difficult tracheostomies safely.

## Introduction

Tracheostomy is commonly performed to relieve respiratory distress, secure definitive airway, and secretion clearance in patients with laryngeal growth, upper respiratory obstruction, trauma, acute respiratory failure, etc. [1]. The trachea anatomically lies in the midline and is easily palpable owing to its superficial location and tracheal rings. As a relatively mobile structure, pathologies along the route can shift the structure and trachea, both in the neck and the thoracic cavity [2]. Tracheostomy becomes technically challenging when the trachea in the neck portion deviates or cannot be palpated, as in obese patients, patients with thyroid malignancy, or surgical emphysema. Gross mediastinal shift and unilateral lung hypoplasia can also lead to tracheal shift right from the origin [3]. In such situations with gross deviations, airway management is tricky and challenging [4]. Surgical tracheostomy is often tricky and tentative and instead can be termed an “exploratory tracheostomy.” Imaging modalities such as computed tomography (CT) scans and magnetic resonance imaging (MRI) can identify the location of the trachea, but the information provided is not real time. However, point-of-care ultrasound (PoCUS) has been utilized as real-time imaging aid for airway management and localizing tracheal rings for tracheostomy [5]. We present two cases in two scenarios with tracheal deviation who underwent either open or percutaneous tracheostomy with the aid of PoCUS and transillumination to delineate the course of the trachea and facilitate difficult tracheostomies safely.

## Case presentation

Case-1

**Figure 1:**
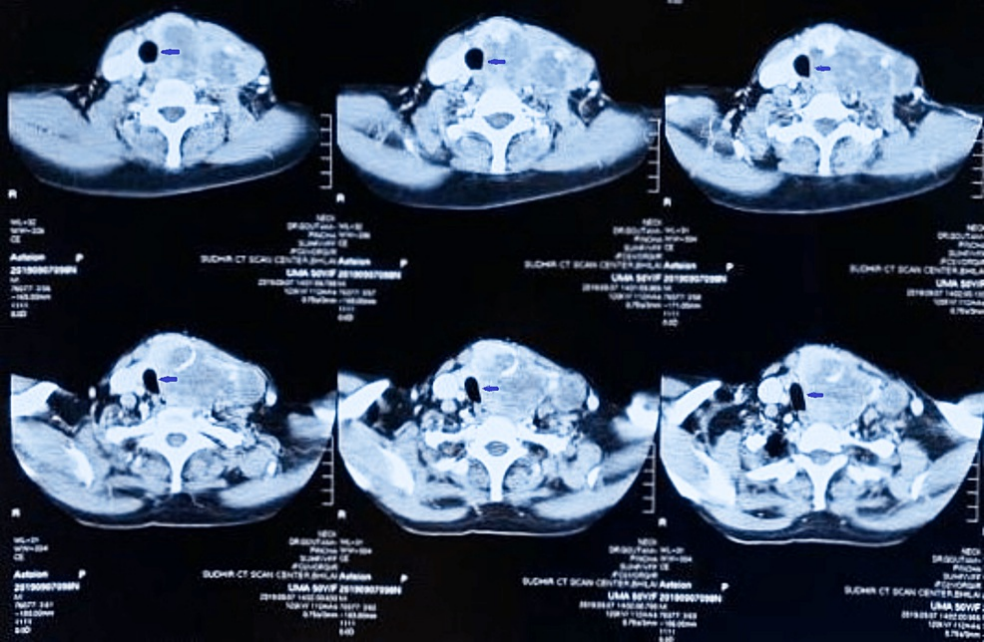
Axial computed tomography scan of the neck showing nonhomogeneous cystic (hypodense) nodules of varied sizes in the front part of the neck, more in the left side (thyroid swelling), and displacement of tracheal air shadow (black shadow pointed by an arrow) from the midline.

A 35-year-old female presented with worsening respiratory distress for seven days and a gradually progressive neck swelling for two years; the distress increased in the supine position. The patient assumed a tripod-like posture to aid in breathing. Her pulse rate was 82 beats/min, regular; blood pressure, 110/70 mmHg; RR, 21 breaths/min; and saturation 97% on room air. The swelling was insidious in onset, diffuse, bilateral, had ill-defined borders, and was firm-hard in consistency. On clinical examination, the trachea could not be located on palpation. CT examination performed earlier elsewhere showed grossly deviated trachea to the right side (Figure 1). Given imminent airway compromise, the patient was posted for emergent surgical tracheostomy.

**Figure 2:**
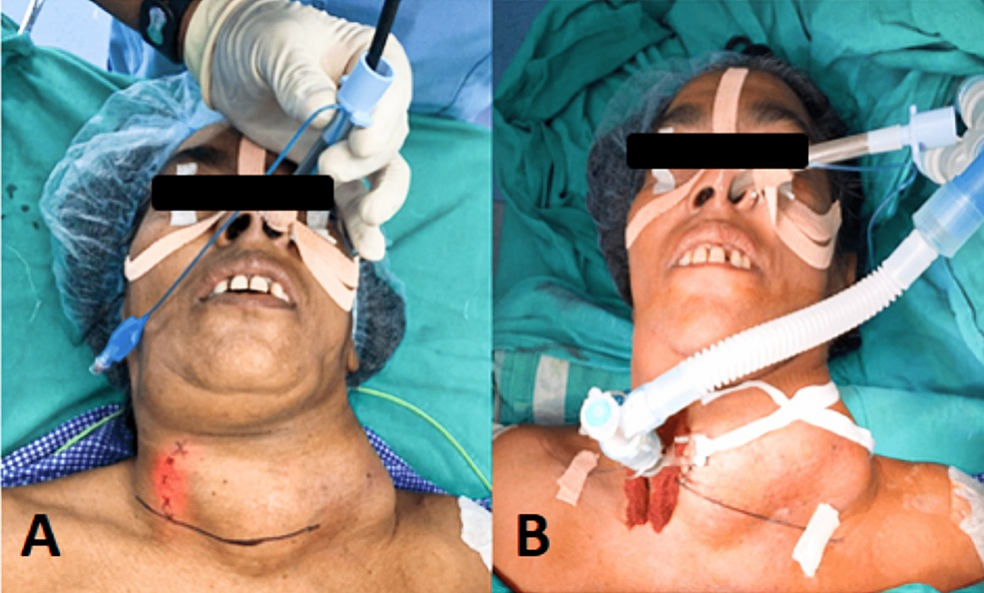
(A) The transilluminated trachea marked with a dotted line. (B) The tracheostomy in situ with a connected circuit.

Moreover, since the patient was restless and could not lie supine, the anesthesiologist and the operating surgeon decided to perform a tracheostomy under general anesthesia (GA). The trachea was intubated under local anesthesia with the help of a video bronchoscope in the sitting position. GA was induced with propofol 100 mg and succinylcholine 100 mg, and the patient was placed in a supine position with the head extended for tracheostomy. Anesthesia was maintained with oxygen:nitrous oxide (50:50) and sevoflurane at MACage 1.2. After that, a video bronchoscope was inserted in the endotracheal tube and advanced gradually to delineate the course of the trachea (Figure 2). A surgical tracheostomy was performed by dissecting the incision over the trachea demarcated during video bronchoscopy. A 7.0 mm cuffed tracheostomy tube (Romsons Scientific & Surgical Pvt. Ltd., Agra, India) was placed, and the position was confirmed. The procedure remained uneventful, and the patient was transferred to the recovery room.

Case-2

**Figure 3:**
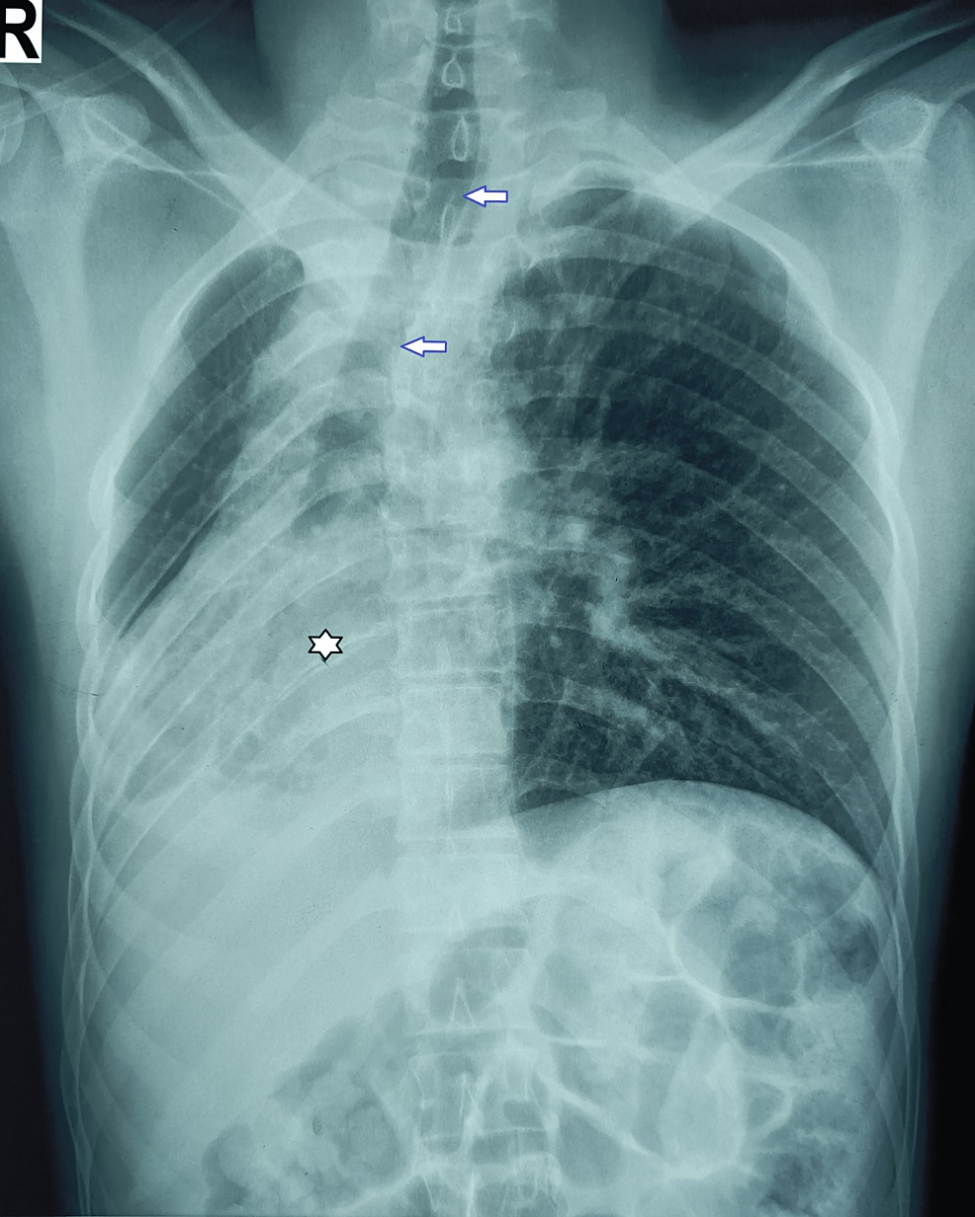
Chest X-ray, including the neck showing the tracheal deviation, mediastinal shift (arrows), and heart shadow in the right hemithorax (star mark).

A 27-year-old male with no active comorbidities met with a road traffic accident and sustained tibia and femur fractures. No history of unconsciousness, nose and ear bleed, seizures, alcohol intake, or other substance and drug abuse was found. He got primary treatment in a local hospital and remained home afterward. The family member gave a history of past pulmonary tuberculosis during his childhood, for which he had to take antitubercular therapy for three years. After nearly 36 hours of injury, he was brought to our hospital with shortness of breath, desaturation, and subsequent altered mentation and irritability. Fat embolism syndrome was suspected; he was intubated to secure his airway and ventilated and was being investigated to find out the etiology. His chest X-ray showed a hyperinflated left lung, a mediastinal shift with heart shadows in the right hemithorax, and a hypoplastic right lung; the trachea was grossly deviated to the right starting from the neck segment (Figure 3).

**Figure 4:**
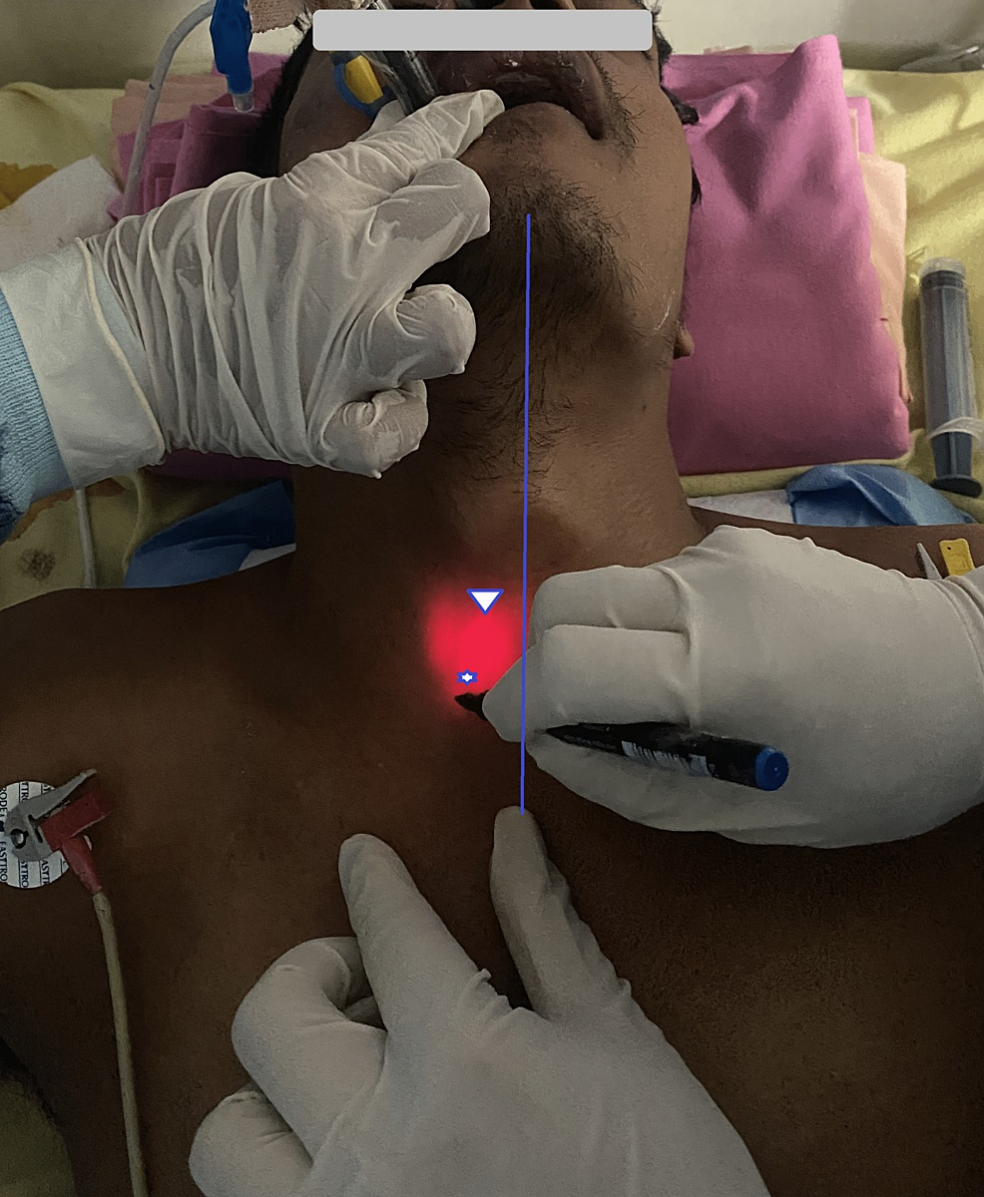
The tracheal transillumination (light in the neck), the cricothyroid membrane (triangle mark), and the puncture point as delineated by PoCUS (star mark) and their relation to the midline (indigo line). PoCUS, point-of-care ultrasound

The critical care team planned an early tracheostomy. Informed consent was obtained from the guardian. As our routine practice, pre-procedural front-of-neck PoCUS was performed to find the midpoint of the tracheal image, puncture point, and any overlying vessel. PoCUS, in this case, showed the relation of the probe marker and neck midline as deviated toward the right. After marking the entry points, it was also decided to use the transillumination technique using fiberscope as described in the first case, which showed the illumination deviated toward the right from the midline (Figure 4). Percutaneous dilatational tracheostomy was done following standard technique, the position was confirmed, and ventilation started without any notable complications.

## Discussion

The trachea is a midline structure, and in most patients, it can be easily palpated, and its course can be defined [2]. Surgical airway access forms the ultimate step in any difficult airway algorithm. Tracheostomies are among the most performed procedures in the operating room and a critical care unit. The procedure has its limitations and complications. In the presence of hyperplasia or hypertrophy of the thyroid, hematoma, surgical emphysema, vascular tumor, thyroid malignancy, and obesity, the trachea is often not palpable and may be impossible to delineate its course on palpation alone [6-8]. Imaging modalities such as CT scans or MRI can delineate its course and help formulate a surgical plan, but the information is not real time. In the present case report, we describe video bronchoscopy for delineating the course of the trachea. Identifying the course of the trachea was precise and helped the operating surgeon to decide on the surgical incision, and the tracheostomy was performed in the usual manner. In a few instances, the author has witnessed surgical tracheostomy becoming “exploratory tracheostomy” in similar circumstances and strongly advocates using this simple airway management technique. Earlier, Addas et al. [9] and Boran et al. [10] described lightwand use to guide intratracheal puncture for percutaneous tracheostomy.

Furthermore, Goneppanavar et al. [11] described lightwand use in four cases for rapidly locating the trachea during a tracheostomy complicated by distorted anatomy. The technique of delineating the trachea using a video bronchoscope is like a lightwand but provides detailed real-time navigation from outside and within the lumen (for percutaneous tracheostomies) and does not interfere with the ventilation or the surgical field [12]. Additionally, Shen et al. [13] have suggested that percutaneous tracheostomies done with external laser light transillumination instill confidence in the surgeon. Despite describing transillumination in literature with various devices, this simple technique has yet to become a common practice inside the operating room, especially during a crisis, as described above.

## Conclusions

The light source of the video bronchoscope or fiberscopes and PoCUS can mark the trachea in cases where it is otherwise not palpable or deviated. Tracheal illumination can help in dissection during tracheostomy in real time while continuing ventilation. Video bronchoscope is frequently available in the anesthetist’s airway armamentarium and can help mark the incision for challenging tracheostomies.
